# BELB: a biomedical entity linking benchmark

**DOI:** 10.1093/bioinformatics/btad698

**Published:** 2023-11-17

**Authors:** Samuele Garda, Leon Weber-Genzel, Robert Martin, Ulf Leser

**Affiliations:** Computer Science Department, Humboldt-Universität zu Berlin, Berlin 10099, Germany; Center for Information and Language Processing, Ludwig-Maximilians-Universität München, München 80539, Germany; Computer Science Department, Humboldt-Universität zu Berlin, Berlin 10099, Germany; Computer Science Department, Humboldt-Universität zu Berlin, Berlin 10099, Germany

## Abstract

**Motivation:**

Biomedical entity linking (BEL) is the task of grounding entity mentions to a knowledge base (KB). It plays a vital role in information extraction pipelines for the life sciences literature. We review recent work in the field and find that, as the task is absent from existing benchmarks for biomedical text mining, different studies adopt different experimental setups making comparisons based on published numbers problematic. Furthermore, neural systems are tested primarily on instances linked to the broad coverage KB UMLS, leaving their performance to more specialized ones, e.g. genes or variants, understudied.

**Results:**

We therefore developed BELB, a biomedical entity linking benchmark, providing access in a unified format to 11 corpora linked to 7 KBs and spanning six entity types: gene, disease, chemical, species, cell line, and variant. BELB greatly reduces preprocessing overhead in testing BEL systems on multiple corpora offering a standardized testbed for reproducible experiments. Using BELB, we perform an extensive evaluation of six rule-based entity-specific systems and three recent neural approaches leveraging pre-trained language models. Our results reveal a mixed picture showing that neural approaches fail to perform consistently across entity types, highlighting the need of further studies towards entity-agnostic models.

**Availability and implementation:**

The source code of BELB is available at: https://github.com/sg-wbi/belb. The code to reproduce our experiments can be found at: https://github.com/sg-wbi/belb-exp.

## 1 Introduction

The task of assigning entity mentions found in biomedical text to a knowledge base (KB) entity is known as biomedical entity linking (BEL). (In some studies “entity linking” denotes the joint entity recognition and linking process. We however refer exclusively to the grounding step. The task is also known as Named Entity Normalization and we will use “linking,” “grounding,” and “normalizing” interchangeably throughout the text.) Texts in the biomedical domain are rich in ambiguous expressions, with abbreviation being a prominent example, e.g. “WSS” can be either “Wrinkly skin syndrome” or “Weaver–Smith syndrome.” BEL resolves such ambiguities and is therefore a crucial component in many downstream applications. For instance, it is used to index PubMed (Mork *et al.* 2013), a primary archive of biomedical literature.

Although several benchmarks have been developed for biomedical text mining, e.g. BLUE (Peng *et al.* 2019) and BLURB (Gu *et al.* 2021), BEL is notably absent from all of them. GeneTuring (Hou and Ji 2023) contains a module to test normalization, but covers only genes and is specific for models built on the GPT-3 architecture. The lack of a standardized evaluation setup translates into a wide variety of approaches: different studies use different combinations of corpus and KB and different evaluation protocols. These differences limit severely direct comparison of results (see Supplementary Data A).

In the biomedical domain, different entity types require normalization to different specialized KBs (Wei *et al.* 2019), e.g. species to NCBI Taxonomy (Scott 2012) but genes to NCBI Gene (Brown *et al.* 2015). Yet, important types such as genes and variants are completely absent from corpora commonly used to evaluate neural BEL approaches (see Section 2.1.1), which instead only target UMLS. Although adapting neural approaches to other KBs is possible, it leaves open the question of whether their performance transfers across entity types. Additionally, as corpora are distributed in different formats, developing new BEL approaches (or adapting existing ones to new corpora) requires writing new input–output and quality assurance routines, e.g. to correct wrong mentions boundaries, increasing the overall engineering turnaround.

**Figure 1.**
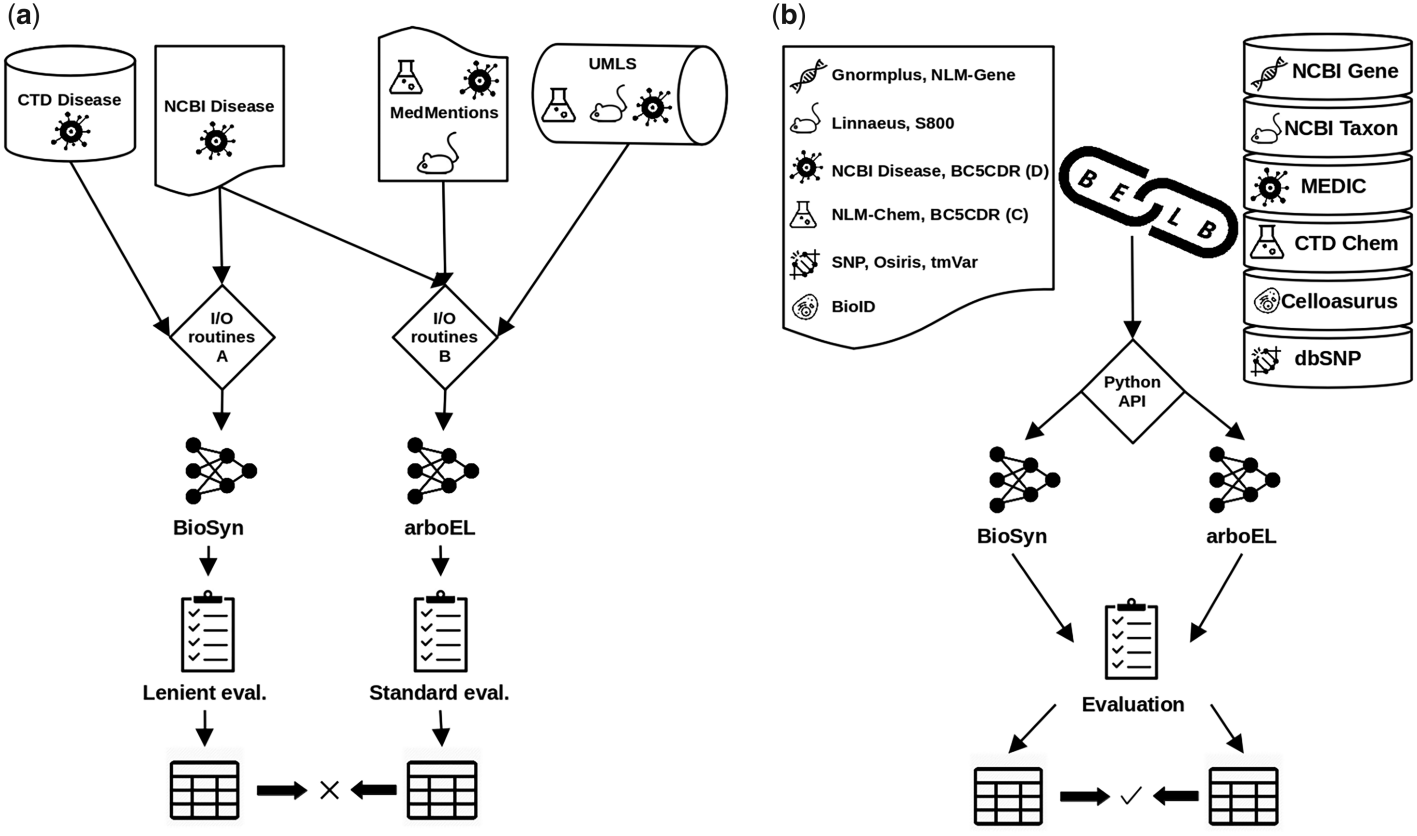
We illustrate the main advantages of BELB. In (a), we see the current stand of experimental setups for BEL. Different studies use different (i) preprocessing (I/O routines), (ii) combinations of corpora and KBs, and (iii) evaluation protocols, ultimately making published numbers not directly comparable. With BELB (b), researcher have access to (i) uniformly preprocessed corpora and KBs, which can be accessed programmatically and (ii) a standardized evaluation protocol greatly reducing preprocessing overhead and maximizing comparability and reproducibility.

To facilitate research on BEL, we introduce BELB, a biomedical entity linking benchmark. BELB provides access to 11 corpora linked to 7 KBs. All components undergo extensive data cleaning and are converted in a “unified format,” covering six biomedical entities (gene, disease, chemical, species, cell lines, and variants). As show in Fig. 1, BELB significantly lowers the barrier for research in the field, allowing to (i) train models on corpora in the highest quality possible and (ii) fairly compare them against other approaches with minimal preprocessing overhead (see Supplementary Data B for a simple showcase). Using BELB, we perform an extensive comparison of “six rule-based domain-specific systems and three neural methods.” Our findings show that results of neural approaches do not transfer across entity types, with specialized rule-based systems still being the overall (Section 4) best option on entity types not explored by neural approaches, namely genes and variants. We hope that our publicly available benchmark will be adopted by future work allowing to fairly evaluate approaches and accelerate progress toward more robust neural models.

## 2 Materials and methods

In this study, we introduce BELB, a benchmark for standardized evaluation of models for BEL. The task is formulated as predicting an entity e∈E from a KB given a document *d* and an entity mention *m*, a pair of start and end positions $\langle m_{s},m_{e}\rangle$ indicating a span in *d*. We use BELB to compare rule-based domain-specific systems and state-of-the-art neural approaches. In all experiments, we use in-KB gold mentions: each mention has a valid gold KB entity (Röder *et al.* 2018) and its position in *d* is given.

### 2.1 Biomedical Entity Linking Benchmark

We report an overview of the 11 corpora and 7 KBs available in BELB in Tables 1 and 2, respectively. Their detailed description can be found in Supplementary Data C. In the following, we outline crucial properties of BEL and highlight how they are accounted for in BELB, allowing it to comprehensively analyse and fairly evaluate BEL models.

**Table 1.** Overview of the corpora available in BELB with their primary characteristics: number of documents, annotations, and how many of them are zero-shot by entity or by name.

	Documents (train/dev/test)	Annotations (train/dev/test)	0-shot entity	0-shot name
Disease				
NCBI disease (Doğan *et al.* 2014)	592/100/100	5133/787/960	150 (15.62%)	185 (19.27%)
BC5CDR (D) (Li *et al.* 2016)	500/500/500	4149/4228/4363	388 (8.89%)	765 (17.53%)
Chemical				
BC5CDR (C) (Li *et al.* 2016)	500/500/500	5148/5298/5334	1038 (19.46%)	415 (7.78%)
NLM-Chem^a^ (Islamaj *et al.* 2022)	80/20/50	20 796/5234/11514	3908 (33.94%)	1534 (13.32%)
Cell line				
BioID^b^ (Arighi *et al.* 2017)	231/59/60	3815/1096/864	158 (18.29%)	45 (5.21%)
Species				
Linnaeus^a^ (Martin *et al.* 2010)	47/17/31	2115/705/1430	385 (26.92%)	58 (4.06%)
S800 (Pafilis *et al.* 2013)	437/63/125	2557/384/767	363 (47.33%)	107 (13.95%)
Gene				
GNormPlus (Wei *et al.* 2015)	279/137/254	3015/1203/3222	2822 (87.59%)	163 (5.06%)
NLM-Gene (Islamaj *et al.* 2021)	400/50/100	11 263/1371/2729	1215 (44.52%)	353 (12.94%)
Variant				
SNP (Thomas *et al.* 2011)	–/–/292	–/–/517	–	–
Osiris v1.2 (Furlong *et al.* 2008)	–/–/57	–/–/261	–	–
tmVar v3 (Wei *et al.* 2022)	–/–/214	–/–/1018	–	–
UMLS				
MedMentions (Mohan and Li 2019)	2635/878/879	122 178/40 864/40 143	8167 (20.34%)	7945 (19.79%)

a Full text.

b Figure caption.

**Table 2.** Overview of the KBs available in BELB according to their entity type.^a^

	Version	History	Entities	Names	Synonyms	Homonyms (PN)
Disease						
CTD diseases (Davis *et al.* 2023)	Monthly^b^	✗	13 188	88 548	6.71	0.39% (–)
Chemical						
CTD chemicals (Davis *et al.* 2023)	Monthly^b^	✗	175 663	451 410	2.56	– (–)
Cell line						
Cellosaurus (Bairoch 2018)	–	✓	144 568	251 747	1.74	3.21% (1.22%)
Species						
NCBI Taxonomy (Scott 2012)	–	✓	2 491 364	3 783 882	1.51	0.04% (–)
Gene						
NCBI Gene (Brown *et al.* 2015)	–	✓	42 252 923	105 570 090	2.49	47.37% (8.32%)
GNormPlus subset			703 858	2 455 772	3.48	50.79% (9.13%)
NLM-Gene subset			873 015	2 913 456	3.33	53.61% (9.55%)
Variant						
dbSNP (Sherry *et al.* 2001)	Build 156^b^	✓	1 053 854 063	3 119 027 235	2.95	1 557 105 418 (49.92%)
UMLS						
UMLS (Bodenreider 2004)	2017AA (full)	–	3 464 809	7 938 833	2.29	2.07% (0.16%)

a We report the number of entities, synonyms per entity, homonyms and how many of them are the primary name (PN).

b No archive of previous versions is provided.

#### 2.1.1 Specialized knowledge bases

In biomedical information extraction, instances of multiple entity types are linked to specialized KBs (Wei *et al.* 2019). However, recent studies in the NLP community primarily focus on the MedMentions corpus linking to UMLS (Liu *et al.* 2021, Agarwal *et al.* 2022 inter alia, Zhang *et al.* 2022). Additionally, in MedMentions, entity types such as diseases and genes are covered only marginally or not at all, respectively (see Supplementary Data D).

This calls into question how well results obtained in this setting can be transferred for instance to publications in genomics or molecular biology in general. In BELB, we cover six entity types (gene, species, disease, chemicals, cell line, and variant) represented by 11 corpora linked to 7 specialized KBs (for comparison with previous studies, we include UMLS as well). We design a “unified schema” to harmonize all KBs (see Supplementary Data E). This allows to test a model’s ability to preserve performance across multiple pairs of corpus and KB with minimal preprocessing overhead.

#### 2.1.2 Zero-shot entities and names

Corpora typically cover only a small fraction of all entities in a KB. Additionally, biomedical entities present multiple names (“synonyms”), e.g. both “Cleidocranial Dysplasia” and “Marie-Sainton Syndrome” are valid names of MeSH: D002973. Hence, if an entity is in the training set, it does not imply that all its surface forms are included. In BELB, we assign a unique identifier to each mention. We then precompute two lists of mentions from all BELB corpora. One with mentions of “zero-shot entities,” i.e. those present in the test set but not in the train one, the other with “zero-shot names” (Tutubalina *et al.* 2020), i.e. train entities occurring in the test set but with different (case-insensitive). This allows to easily report a model’s average performance in (i) generalizing to new entities and (ii) recognizing known ones appearing in different forms.

#### 2.1.3 Homonyms

Discriminating mentions with the same surface form but representing different entities (“homonyms”) by their context is indispensable to BEL. This is because in biomedical KBs, the same synonym can be associated to multiple entities. This is especially the case of abbreviations. For instance, as in example (a) in Table 3, “WSS” is the abbreviated form of two syndromes and it appears twice in CTD Diseases. Another example is genes present in multiple species, as in (c), where the string “rats” is essential for correct normalization, as “α2microglobulin” could refer either to the human or rat gene. Identifying contextual information can be non-trivial, e.g. (c) is the title of a publication, but the text may describe general characteristics of “α2microglobulin” introducing textual cues pointing to the human gene. Additionally, this information is not always explicitly expressed and may emerge via other patterns. In example (e), “PC12” denotes a human cell line, whereas in (f), it refers to the rat one. This can be inferred from the capitalized gene mention “SGK1” in (e) which conventionally denotes human genes. By introducing entity types such as genes and variants, BELB allows to probe a model’s ability to (i) identify contextual information and (ii) handle highly ambiguous search spaces (KB).

**Table 3.** Example of homonym mentions (in bold) requiring specific contextual information (underlined) for linking.

	Example	Entity
(a)	Features of ARCL type II overlap with those of Wrinkly skin syndrome (**WSS**).	MeSH: C536750
(b)	Weaver–Smith syndrome (**WSS**) is a Mendelian disorder of the epigenetic machinery.	MeSH: C536687
(c)	α **2microglobulin** exacerbates brain damage after stroke in rats.	NCBI Gene: 24153
(d)	The T67 cell line produced the proteinase inhibitor α**2microglobulin**.	NCBI Gene: 2
(e)	We identified the novel mutation **c.908G**>**A** within exon 8 of the CTSK gene.	rs756250449
(f)	The patient was compound heterozygous of the **c.908G**>**A** mutation in the SLC17A5 gene.	rs1057516601
(g)	The GSK650394 inhibitor is used to suppress SGK1 expression in **PC12 cells**.	CVCL_S979
(h)	Effects of topography on rat pheochromocytoma cell, **PC12 cells**, neuritogenesis.	CVCL_0481

#### 2.1.4 Scale

As mentioned in Section 2.1.1, studies on neural methods have primarily targeted UMLS, which, as shown in Table 2, is one and three order of magnitude(s) smaller than NCBI Gene and dbSNP, respectively. With its unified format, BELB allows to easily test how implementations scale to these large KBs.

#### 2.1.5 Synchronization of KB versions

Entity linking is dynamic by nature: over the years, entities in KBs are replaced or become obsolete. For instance, in GNormPlus mentions of “MDS1” are linked to NCBI Gene entity “4197,” which was subsequently replaced by “2122.” As several KBs do not have a versioning system (see Table 2), it is often not possible to retrieve the exact KB used to create a corpus. Failing to account for these changes may introduce a notable amount of noise in measuring performance of high quality systems. If available, BELB integrates in its API the KB “history,” i.e. a table tracking all changes in the entities. This allows to automatically update all corpora with the KB version at hand and remove mentions linked to obsolete entities. It allows as well to update the predictions of systems shipping with a pre-processed (i.e. non-trivially swappable) KB on corpora created after their release, allowing for fair comparison “over time.”

### 2.2 Evaluated approaches

We use BELB to perform an extensive evaluation of rule-based and neural methods. We now present the selected approaches for evaluation. We stress that we do not re-implement any method (we rely on the original code).

#### 2.2.1 Rule-based entity-specific systems

We compare the performance of neural models linking to KBs for which specialized systems have been developed, as these are still the *de facto* standard in BEL (Wei *et al.* 2019, Mujeen *et al.* 2022). Specifically, we test the following rule-based methods: “GNormPlus” (Wei *et al.* 2015) for genes (NCBI Gene), “SR4GN” (Wei *et al.* 2012) for species (NCBI Taxonomy) and “tmVar v3” (Wei *et al.* 2022) for variants (dbSNP). For UMLS, we employ “SciSpacy” (Neumann *et al.* 2019). We label them rule-based entity-specific (RBES) as for linking they rely on a mixture of string matching approaches and *ad hoc* rules tailored to a specific entity type. For diseases and chemicals, we include in the RBES category two systems which are only “partly” rule-based (stretching our definition), as they better represent the state of the art of disease/chemical-specific models. We use “TaggerOne” (Leaman and Lu 2016), a semi-Markov model, for diseases, and opt for the system that won the BioCreative VII NLM-Chem track (Almeida *et al.* 2022) for chemicals (“BC7T2W”), which uses both string matching and neural embeddings. To the best of our knowledge there exists no linking approach specific for cell lines. We therefore use a fuzzy string matching approach based on Levenshtein distance (“FuzzySearch”). For detailed descriptions and information on specific implementations, we refer the reader to Supplementary Data F.1. All of these systems do not require re-training as either (i) their normalization component is completely rule-based (SR4GN, tmVar, SciSpacy) or (ii) models trained on the BELB corpora are provided along with the code (GNormPlus, TaggerOne, BC7T2W).

#### 2.2.2 Neural systems

We train from scratch the following neural models on BELB (see Supplementary Data F.2 for training details). To ensure that all systems receive the same training signal, we avoid corpus- or KB-specific pre-trained weights.

“BioSyn” (Sung *et al.* 2020) is a dual encoder architecture. Importantly, BioSyn does not account for context, i.e. it uses only entity mentions. The model is trained via “synonym marginalization”: it learns to maximize the similarity (inner product) between a mention embedding and all the synonyms embeddings of the gold entity. At inference, it retrieves the synonyms most similar to the given test mention, i.e. it relies on a lookup from synonym to entity. We prefer BioSyn over SapBERT (Liu *et al.* 2021) as the latter is primarily a pre-training strategy.

“GenBioEL” (Yuan *et al.* 2022) is an encoder–decoder model. As input, it takes a text with a single mention marked with special tokens. The system is then trained to generate a synonym. At inference, it ensures that the prediction is a valid KB synonym by constraining the generation process with a prefix-tree created from all KB synonyms. Similar to BioSyn, this approach represents KB entities by their synonyms. The authors propose as well “KB-Guided Pre-training,” i.e. a method based on prompting to pre-train GenBioEL on the KB. We avoid this step because (i) we exclude pre-training strategies (see above) and (ii) it is computationally expensive: it would require to pre-train GenBioEL on all seven KBs.

“arboEL” (Agarwal *et al.* 2022) is a dual encoder as well. The authors propose to construct *k*-nearest neighbor graphs over mention and entity clusters. Using a pruning algorithm, they then generate directed minimum spanning trees rooted at entity nodes, and use the edges as positive examples for training. At inference, they use the entity present in the mention’s cluster. Notably, arboEL learns “entity embeddings,” encoded as a concatenated list of synonyms. The authors train as well a cross-encoder (Wu *et al.* 2020), i.e. a second model reranking the entities retrieved by a trained arboEL. We avoid this extension as it is not strictly part of the arboEL algorithm.

### 2.3 Evaluation protocol

We now describe in detail the evaluation protocol which we followed in our experiments. For all systems, we report the micro-average mention-level recall@1 (accuracy), since RBES approaches generate only a single candidate.

#### 2.3.1 Synonym as entity proxy

Approaches using strings as proxies for entities (BioSyn, GenBioEL) cannot meaningfully resolve ambiguous mentions. That is, for a mention of rat “α2microglobulin,” they would return a list containing “both” NCBI Gene “2” (human) and “24153” (rat). Sung *et al.* (2020) introduced a “lenient” evaluation, which considers a prediction correct if any of the returned entities matches the gold one. As reported by Zhang *et al.* (2022), this largely overestimates performance. Following their suggestion, we opt for a “standard” evaluation, which randomly samples one prediction from the returned list. However, as one of the aims of BEL is direct deployment in extraction pipelines, e.g. for constructing gene networks (Lehmann *et al.* 2015), we also include a *strict* evaluation in which all such cases, i.e. multiple predictions, are considered incorrect.

#### 2.3.2 Disentangling recognition and linking

Some RBES systems (GNormPlus, SR4GN, TaggerOne, tmVar) perform entity recognition and linking jointly. Due to false negatives in the NER step, we cannot obtain their results on the full test set. To ensure that we are measuring the performance on the same instances for all methods, for corpora whose reference RBES system is a joint approach, we use only the test mentions which are correctly identified during entity recognition. For instance, for NLM-Gene, we use only 73% of the test mentions, i.e. those correctly recognized by GNormPlus. As correct recognition correlates with correct normalization, our evaluation protocol probably introduces a bias toward RBES systems (see Section 4).

#### 2.3.3 Multiple gold entities

Mentions in biomedical corpora can provide multiple normalizations. Common instances are “composite mentions,” e.g. “breast and squamous cell neoplasms” and ambiguous ones, e.g. “Toll-like receptor” (“Toll-like receptor 2,” “4,” and “9”). Whether these cases are logical AND or is not always specified in the annotation guidelines. We opt for the more lenient OR interpretation and consider a prediction correct if it matches one of the gold entities.

## 3 Results

Table 4 reports the results of neural models and entity-specific models (grouped under the RBES category) on the filtered (Section 2.3.2) BELB test set with standard evaluation (see Supplementary Data G for additional evaluations). We observe that performance of neural models varies significantly across entity types, with gene corpora incurring the most significant drop.

**Table 4.** Performance of all baselines on filtered (Section 2.3.2) BELB test set.^a^

Entity type				
Corpus	RBES	BioSyn	GenBioEL	arboEL
Disease	0.94	0.87	0.90	0.87
NCBI Disease	0.94 (FN:173/960, FP:156)	0.83	0.89	0.86
BC5CDR (D)	0.94 (FN:781/4363, FP:744)	0.88	0.92	0.88
Chemical	0.72	0.72	0.81	0.77
BC5CDR (C)	0.82	0.85	0.95	0.88
NLM-Chem	0.67	0.67	0.75	0.72
Cell line				
BioID	0.82	0.82	0.96	0.95
Species	0.97	0.91	0.85	0.76
Linnaeus	0.99 (FN:291/1430, FP:199)	0.93	0.81	0.74
S800	0.93 (FN:231/767, FP:215)	0.88	0.93	0.79
Gene	0.82	–	0.17	0.30
GNormPlus	0.87 (FN:212/3222, FP:201)	OOM	0.21	0.36
NLM-Gene	0.76 (FN:746/2729, FP:102)	OOM	0.13	0.23
Variant	0.91	–	–	–
SNP^a^	0.94 (FN:44/517, FP:5149)	–	–	–
Osiris v1.2	0.91 (FN:85/787, FP:787)	–	–	–
tmVar v3	0.88 (FN:81/1018, FP:2906)	–	–	–
UMLS				
MedMentions	0.58	OOM	0.57	0.69

a Performance of all models on the BELB benchmark (test set). All scores are mention-level recall@1. For joint recognition and normalization RBES we report the amount of NER false negatives (FN) and false positives (FP). OOM: out-of-memory (>200GB)

**Homonyms:** Beside the implicit bias toward RBES approaches (see Section 2.3.2), we hypothesize that an important factor at play are homonyms. RBES systems use *ad hoc* components to handle these challenging cases. For instance, GNormPlus directly integrates Ab3P (Sohn *et al.* 2008), a widely adopted abbreviation resolution tool, and SR4GN, which is specifically developed for cross-species gene normalization. Neural models lack these components, and synonym-based approaches are significantly impacted by random selection in case of homonyms. In Table 5, we show that if we deploy a lenient evaluation (Section 2.3.1), results on each corpus improve proportionally to the amount of homonyms in the corresponding KB. Results with expanded abbreviations indicate that in the case of NCBI Disease, many homonyms are abbreviations, while in GNormPlus this is not the case. In contrast, abbreviation resolution has no impact on arboEL. We argue that this is due to the fact that arboEL uses entity embeddings, i.e. it cannot perform direct string matching, thus benefiting less by expanded abbreviations. Secondly, as entity embeddings require to learn a compressed entity representation, arboEL is affected by the limited size of the corpora. This is supported by results on MedMentions, which is one order of magnitude larger than other BELB corpora, where arboEL is confirmed the state-of-the-art approach.

**Table 5.** Relative improvement of neural models with resolved abbreviations and a lenient evaluation in case of multiple “predicted entities.”^a^

	NCBI Disease	GNormPlus
RBES	0.94	0.87
BioSyn	0.83	OOM
+ abbr. res.	0.88 (+0.5%)	–
+ lenient eval.	0.88 (+0.5%)	–
GenBioEL	0.89	0.21
+ abbr. res.	0.91 (+0.2%)	0.20 (−0.1%)
+ lenient eval.	0.90 (+0.1%)	0.86 (+65%)
arboEL	0.86	0.36
+ abbr. res.	0.86 (–)	0.36 (–)
+ lenient eval.	–	–

a OOM: out of memory (>200GB).

**Zero-shot entities and names:** In Table 6, we see that neural approaches are outperformed by RBES systems on mentions of unseen entities while the opposite happens with unseen names of train entities. This can be explained by the fact that as string-matching approaches have direct access to the KB they are better suited for the zero-shot cases. If training data are available, neural representation is superior instead, as they can leverage representations learned from context.

**Table 6.** Results on zero-shot entities and names (mention-level recall@1).

	0-shot entity	0-shot name
RBES	0.65	0.40
GenBioEL	0.50	0.48
arboEL	0.45	0.50

**Scale:** Neural models implementations fail to scale to large KBs such as NCBI Gene or dbSNP. In our experiments, we resorted to use the NCBI Gene subset determined by the species of the entities found in the gene corpora (see Table 2). This reflects a common real-world use case scenario, since often only a specific subset of species is relevant for linking (e.g. human and mouse). For dbSNP, we are not aware of a valid criterion to subset it and we are therefore unable to run neural models on variants corpora.

**Synchronization of KB version:** Corpora are only sparsely affected by changes in entities. However, if they are not handled properly, in BC5CDR (C) and Linnaeus (the most affected corpora in BELB), even if evaluating a perfect system, we would register an error rate of 4.56% and 3.57%, respectively (see Supplementary Data H).

## 4 Discussion

We strived to include in BELB as many corpora and KBs as possible, prioritizing those which are most commonly used by the community. We leave as further improvement the expansion to other important research directions as applications to clinical notes (Luo *et al.* 2020) and other languages as Spanish (Miranda-Escalada *et al.* 2022) and German (Kittner *et al.* 2021).

Our evaluation presents a mixed picture. RBES systems are still superior in several instances, especially on genes. However, importantly, we consider only the test mentions they correctly identify (Section 2.3.2). Tough, we argue, this is the best approximation to fairly compare results across methods, it introduces a bias toward these systems. Secondly, by not allowing to decouple recognition and linking, RBES systems are less appealing in applications where only the latter is required as they introduce NER false positives (see Table 4). Finally, RBES systems are advantaged by the use of *ad hoc* components to handle homonyms. In Table 5, we show that introducing similar approaches for neural models could significantly improve their performance. However, as the neural paradigm is based on learning task-related capabilities from data (Bengio *et al.* 2013), we believe that future studies should nevertheless continue to investigate entity-agnostic models, rather than falling back to custom-made entity-specific heuristics.

Neural approaches fail to perform consistently across all BELB instances. However, our evaluation excluded enhancements common for these models. Due to the high computational requirements, we do not perform hyperparameter exploration and rely instead on those reported by the authors. For a fair comparison across method, we avoid as well KB-specific pretraining (Liu *et al.* 2021, Yuan *et al.* 2022) and candidates reranking, e.g. with a cross-encoder (Agarwal *et al.* 2022). Finally, current implementations fail to scale to large KBs, making them unusable for some real-world applications. We leave as future work the investigation of approaches combining RBES and neural models. To the best of our knowledge, only BERN2 (Mujeen *et al.* 2022) uses this paradigm which however we exclude as (i) it is limited to genes, disease, and chemicals and (ii) by using different RBES than ours, we cannot directly estimate the impact of the neural addition.

## 5 Conclusion

We presented BELB, a benchmark to standardize experimental setups for BEL. We conducted an extensive evaluation of RBES systems and recent neural approaches. We find that the first, with some caveats (Section 4), are still the state of the art on entity types not explored by neural approaches, namely genes and variants. We hope that BELB will encourage future studies to compare approaches with a common testbed and to address current limitations of neural approaches.

## Supplementary Material

btad698_Supplementary_Data

## Data Availability

The data underlying this article are available at https://github.com/sg-wbi/belb-exp.
