# Supplementary material for: BELB: a biomedical entity linking benchmark
Source: Bioinformatics. 2023 Nov 17;39(11):btad698. doi: 10.1093/bioinformatics/btad698 (PMC10681865; doi:10.1093/bioinformatics/btad698)
Supplement: btad698_Supplementary_Data [file btad698_supplementary_data.pdf]

Journal Title Here, 2022, 1–9
doi: DOI HERE
Advance Access Publication Date: Day Month Year
Paper

Supplementary data for

BELB: a Biomedical Entity Linking Benchmark

Samuele Garda,[1,*] Leon Weber-Genzel,[2] Robert Martin[1] and Ulf Leser[1,*]

[1]Computer Science, Humboldt-Universität zu Berlin, Rudower Chaussee 25, 12489, Berlin, Germany and [2]Center for Information and Language Processing, Ludwig-Maximilians-Universität München, Geschwister-Scholl-Platz 1, 80539, München, Germany

*To whom correspondence should be addressed.

FOR PUBLISHER ONLY Received on Date Month Year; revised on Date Month Year; accepted on Date Month Year

Abstract

Availability: The source code of BELB is available at: https://github.com/sg-wbi/belb. The code to reproduce our experiments can be found at: https://github.com/sg-wbi/belb-exp.

Contact: gardasam@informatik.hu-berlin.de, leser@informatik.hu-berlin.de

A. Neural biomedical entity linking

Model<br>Abbr. res. / Pre-train / Lenient eval.	BC5CDR (Disease / Chemical)	NCBI Disease	MedMentions
BioSyn (Sung et al., 2020)<br>Sohn et al. (2008) / - / yes	93.2 (CTD Diseases) / 96.6 (CTD Chemicals)	91.1 (CTD Diseases)	-
SapBERT (Liu et al., 2021)<br>Sohn et al. (2008) / KB / yes	93.2 (CTD Diseases) / 96.5 (CTD Chemicals)	92.3 (CTD Diseases)	50.4
BertOverkill (Lai et al., 2021)<br>Sohn et al. (2008) / - / yes	93.2 (CTD Diseases) / 96.9 (CTD Chemicals)	92.2 (CTD Diseases)	55.0
Lightweight BEL (Chen et al., 2021)<br>Sohn et al. (2008) / - / no	-	89.59 (CTD Diseases)	-
ClusteringInference (Angell et al., 2021)<br>Sohn et al. (2008) / - / no	91.3 (UMLS) † ‡	-	74.1 ‡
MedWiki (Varma et al., 2021)<br>Schwartz and Hearst (2002) / Wikipedia,PubMed / no	91.5 (UMLS) † ‡	-	74.6 ‡
KRISSBERT (Zhang et al., 2022)<br>- / PubMed / no	90.7 (UMLS) / 96.9 (UMLS) ‡	89.9 (UMLS) ‡	70.6 ‡
GenBioEL (Yuan et al., 2022)<br>Sohn et al. (2008) / KB / yes	92.6 (MeSH) † ◇	91.6 (CTD Diseases) ◇	-
arboEL (Agarwal et al., 2022)<br>- / - / no	-	-	72.31

Table 1. Overview of experimental designs adopted by recent neural approaches for biomedical entity linking. We highlight with a color all cells reporting results which can be compared directly, i.e.: same corpus and knowledge base, abbreviation resolution (Abbr. Res.), pre-training (if any) and evaluation protocol. † No distinction between disease and chemical annotations ‡ Reranking model ◇ Ablation study without pre-training

Studies in BEL have converged to using primarily three corpora: `NCBI Disease` (Doğan et al., 2014), `BC5CDR` (Li et al., 2016) and `MedMentions` (Mohan and Li, 2019). However, as shown in Table 1, where we report a summary of recent approaches, experimental setups differ importantly in terms of the corpora and KBs used, making comparison based solely on published numbers problematic.

© The Author 2022. Published by Oxford University Press. All rights reserved. For permissions, please e-mail: journals.permissions@oup.com

For instance, in BioSyn the BC5CDR corpus is divided into two, distinguishing between chemical and disease entities, and linked to the CTD vocabularies, while GenBioEL reports results on the entire corpus linking to MeSH, preventing direct comparison. Notably, neural approaches rely on different pre-training strategies with different data sources, making the training signal vary significantly across approaches. This ultimately hinders estimating the difference in performance stemming purely from algorithmic differences. For instance MedWiki outperforms KRISSBERT on MedMentions, but as it is pre-trained on a larger pool of documents, it is unclear whether this is due to differences in pre-training or model architecture. Similarly, it is not possible to directly estimate the impact of using abbreviation resolution in the reported performance. Finally, studies differ in the type of evaluation used, with some models deploying a lenient evaluation further hindering direct comparison even if the corpus and the KB are the same.

B. Showcase

Listing 1 Example code to test an exact-match approach on BELB.

```python
from collections import defaultdict(list)
from sqlalchemy import select
from belb import (AutoBelbKb, AutoBelbCorpus,
                  CORPUS_TO_KB, Tables)

from belb.resources import Corpora, Kbs

for corpus in Corpora:
  corpus = AutoBelbCorpus.from_name(corpus.name)

  kb = AutoBelbKb.from_name(CORPUS_TO_KB[corpus.name])

  table = kb.schema.get(Tables.KB)
  query = select(table.c.entity, table.c.name)

  name_to_entity = defaultdict(list)
  with kb as handle:
    for row in hadle.query(query):
      synonym = row["name"].lower()
      name_to_entity[synonym].append(row["entity"])

  for document in corpus["test"]:
    for a in document.annotations:
      name_to_entity.get(a.text.lower(), -1)
```

In Listing 1 we show that with BELB in less than 30 lines of code it is possible to test a simple exact-match approach on all its available pairs of corpus and knowledge base.

C. Corpora and Knowledge Bases

In this section we describe in detail the corpora and knowledge bases contained in BELB, grouped by their entity type:

Gene For genes, in contrast to previous approaches (Tutubalina et al., 2020), we use `GNormPlus` (Wei et al., 2015) instead of the BioCreative II Gene Normalization (BC2GN) corpus (Morgan et al., 2008). This is because BC2GN (i) is limited to human genes and (ii) it provides identifier annotations only at the *document level*. `GNormPlus` consists of two corpora re-annotated at the *mention level*, namely: BC2GN and the Gene Indexing Assistant (GIA) test collection[1]. We devote the GIA test collection as development split since GNormPlus (corpus) does not provide one. We include as well `NLM-Gene` (Islamaj et al., 2021), which covers ambiguous gene names by including more species. As it offers only a train and a test split, we randomly sample 10% (50 documents) of the training data to be used for development. Both corpora are linked to NCBI Gene (Brown et al., 2015), which integrates detailed information for known and predicted genes, including data from all major taxonomic groups.

Disease Standard corpora for disease normalization are `NCBI Disease` (Doğan et al., 2014) and `BC5CDR` (Li et al., 2016), originally created for the Chemical Disease Relation (CDR) track at BioCreative V. Entity mentions in these corpora are normalized to CTD Diseases (Davis et al., 2023), a.k.a. MEDIC, which is a modified subset of descriptors from the "Diseases" branch of MeSH (Lipscomb, 2000), combined with genetic disorders from the OMIM database (Hamosh et al., 2005).

[1] https://ii.nlm.nih.gov/TestCollections/

	Website (link)	License
**Corpora**		
GNormPlus	https://www.ncbi.nlm.nih.gov/research/bionlp/Tools/gnormplus/	Public domain
NLM-Gene	https://zenodo.org/record/5089049	CC0 1.0
NCBI Disease	https://www.ncbi.nlm.nih.gov/CBBresearch/Dogan/DISEASE/	Public domain
BC5CDR	https://biocreative.bioinformatics.udel.edu/tasks/biocreative-v/track-3-cdr/	Public domain
NLM-Chem	https://biocreative.bioinformatics.udel.edu/tasks/biocreative-vii/track-2/	CC0 1.0
Linnaeus	https://linnaeus.sourceforge.net/	N/A
S800	https://species.jensenlab.org/	N/A
BioID	https://biocreative.bioinformatics.udel.edu/tasks/biocreative-vi/track-1/	N/A
MedMentions	https://github.com/chanzuckerberg/MedMentions	CC0 1.0
SNP	http://www.scai.fraunhofer.de/snp-normalization-corpus.html	N/A ‡
Osiris v1.2	http://dx.doi.org/10.1186/1471-2105-9-84 †	CC BY 3.0
tmVar v3	https://www.ncbi.nlm.nih.gov/research/bionlp/Tools/tmvar/	Public domain
**Knowledge bases**		
CTD Diseases	http://ctdbase.org/downloads/#alldiseases	Copyrighted ◇
CTD Chemicals	https://ctdbase.org/downloads/#allchems	Copyrighted ◇
NCBI Taxonomy	https://www.ncbi.nlm.nih.gov/taxonomy	Public domain
Cellosaurus	https://www.cellosaurus.org/	CC BY 4.0
NCBI Gene	https://www.ncbi.nlm.nih.gov/gene	Public domain
dbSNP	https://www.ncbi.nlm.nih.gov/snp/	Public domain
UMLS	https://www.nlm.nih.gov/research/umls/index.html	DUA

Table 2. Corpora in BELB with corresponding link and license information. † The original website is no longer available: https://sites.google.com/site/laurafurlongweb/databases-and-tools/corpora/. The original corpus is redistributed by (Thomas et al., 2016). ‡ Commercial use is forbidden. ◇ Free for non-commercial uses, otherwise requires paid licensing

Chemical For chemicals we use BC5CDR as it provides as well chemical annotations and additionally include NLM-Chem, a collection of *full text* articles. Specifically we employ the version released for the NLM-Chem track at BioCreative VII (Islamaj et al., 2022). Mentions in these corpora are linked to the chemical branch of MeSH, which however includes broad categorises such as "D014867" ("water"). We therefore only retain entity mentions linked to CTD Chemicals (Davis et al., 2023), which merges descriptors from the "Chemicals and Drugs" category and Supplementary Concept Records in MeSH and removes several branches of the original MeSH hierarchy if either (i) they are not molecular reagents/clinical drugs, e.g."Purines", or (ii) they are broad chemical classes, e.g. "Poisons".

Species For species normalization, we use the Linnaeus corpus (Martin et al., 2010), a collection of annotated full text articles, and S800 (Pafilis et al., 2013), containing 100 abstracts from 8 different publication categories (e.g. bacteriology). Both corpora are linked to NCBI Taxonomy (Scott, 2012), the standard nomenclature and classification repository of organisms for sequences databases such as GenBank. Both corpora do not provide canonical splits. We rely on those defined in https://github.com/spyysalo/linnaeus-corpus/tree/master/split and https://github.com/spyysalo/s800 for Linnaeus and S800 respectively.

Cell line To the best of our knowledge, the only available corpus offering normalized entity mentions of cell lines is BioID (Arighi et al., 2017), introduced in the Interactive Bio-ID Assignment Track at BioCreative VI. In contrast to all other corpora, text in BioID are figure captions found in full text articles. The corpus is annotated with mentions of multiple entity types (e.g. chemicals and species), but we retain exclusively those linked to Cellosaurus (Bairoch, 2018), a nomenclature of cell lines used in biomedical research. Only the annotated training split was made available for the BioCreative track. We therefore re-structure the training data into 80/10/10 train, development and test split respectively.

Variant The de facto standard corpora for variants are: SNP (Thomas et al., 2011), Osiris v1.2 (Furlong et al., 2008) and tmVar v3 (Wei et al., 2022). All corpora contain exclusively a test split. The tmVar (v3) corpus offers multiple normalizations for a given entity mention, e.g. a CAR Canonical Allele Identifier (CA ID) or its corresponding gene. For all corpora, we keep only the entity mentions which are normalized to dbSNP, the primary knowledge base for human genetic variants. Despite its name, dbSNP contains a wide range of polymorphisms besides Short Nucleotide Polymorphisms, e.g. short deletion and insertion polymorphisms and multinucleotide polymorphisms.

UMLS For comparison with previous studies we include the MedMentions corpus with the 2017AA full release of UMLS. We note that although CTD Diseases and CTD Chemicals are technically a subset of UMLS, we keep them as separate KBs because (i) some corpora were specifically linked to them (e.g. NCBI Disease) and (ii) with UMLS containing many unrelated concepts, it would unnecessarily increase the search space.

In Table 2 we report links to all resources included in BELB along with the license with which they are released. All resources created by NLM, as a governmental agency, are by nature of public domain[2]. Many corpora do not specify a license (we mark them as

[2] See https://www.ncbi.nlm.nih.gov/CBBresearch/Dogan/DISEASE/disclaimer.html for the public domain notice which usually accompanies these resources.

N/A) but can be freely accessed and do not specify any limitation on the data usage (except in forbidding commercial use). The same considerations hold for the knowledge bases. UMLS is the only resource which is not freely available and users are required to enter a Data Usage Agreement (DUA).

D. MedMentions

	Semantic Group	Entity mentions (%)
1	Phenomena	41,422 (20%)
2	Chemicals & Drugs	37,401 (18%)
3	Procedures	33,478 (16%)
4	Concepts & Ideas	22,824 (11%)
5	Anatomy	22,248 (11%)
6	Living Beings	19,626 (10%)
7	Disorders	18,081 (9%)
8	Organizations	2,143 (1%)
9	Devices	2,018 (1%)
10	Physiology	1,773 (1%)
11	Objects	1,352 (1%)
12	Occupations	9,16 (0%)
13	Activities & Behaviors	0 (0%)
14	Genes & Molecular Sequences	0 (0%)
15	Geographic Areas	0 (0%)

Table 3. Ranked number of entity mentions (and relative amount) in MedMentions (ST21PV) grouped by UMLS Semantic Groups.

In Table 3 we report the ranked number of entity mentions in MedMentions (ST21PV) grouped by their UMLS Semantic Groups[3]. We see that entity types such as diseases ("Disorders") and species ("Living Beings") represent only a small fraction of the overall entity types, while genes ("Genes & Molecular Sequences") are completely absent. Notably, a high proportion of mentions (∼47%) is devoted instead to general entities such as "Phenomena", "Concepts & Ideas" and "Procedures".

E. Unified schema for knowledge bases

Listing 2 Example SQL code defining the BELB schema for all knowledge bases.

```
CREATE TABLE kb(
    uid INTEGER PRIMARY KEY,
    entity INTEGER NOT NULL,
    description INTEGER NOT NULL, -- 0: scientific name, 1: common name, ...
    name TEXT NOT NULL,
    foreign_entity INTEGER REFERENCES foreign_entities(entity),
)

CREATE TABLE foreign_entities(
    entity INTEGER NOT NULL,
    name TEXT NOT NULL,
)

CREATE TABLE history(
    old INTEGER PRIMARY KEY,
    new INTEGER NOT NULL,
)
```

In Listing 2 we provide an overview of the unified schema used to store all knowledge bases in BELB. In its basic form, a KB is a list of synonyms (names), each associated with a single entity. All KBs provide as well information about each name ("description"). For

[3] https://lhncbc.nlm.nih.gov/ii/tools/MetaMap/documentation/SemanticTypesAndGroups.html

instance in NCBI Taxonomy a name can be the "scientific name" of a species ("Homo Sapiens") or the "common" one ("human"). In all KBs exclusively one name must be the primary one, i.e. the one most commonly used to refer to the concept represented by the entity. For instance, the primary name (a.k.a symbol) for entity "2" in NCBI Gene is "A2M". Some of the KBs are interconnected. For instance, "α2microglobulin" represents a different entity when referring to the human or rat gene. Thus, besides having different identifiers ("2" and "24153" respectively), to ease downstream applications (e.g. data integration), NCBI Gene provides what we call (borrowing from the database jargon) a *foreign* entity. For instance, all entries in NCBI Gene with identifier 2 are accompanied by the foreign entity "9606", i.e. the entity in NCBI Taxonomy denoting "Homo Sapiens" (human). If available, a KB provides as well a *history* table, where changes to the identifiers are tracked, i.e. if they have been replaced by others or have become obsolete.

F. Biomedical entity liking systems

	Implementation (link)
**Rule-based entity-specific**	
GNormPlus	https://www.ncbi.nlm.nih.gov/research/bionlp/Tools/gnormplus/
TaggerOne	https://www.ncbi.nlm.nih.gov/research/bionlp/Tools/taggerone/
tmVar v3	https://ftp.ncbi.nlm.nih.gov/pub/lu/tmVar3/
BC7T2W	https://github.com/bioinformatics-ua/biocreativeVII_track2
SR4GN	https://www.ncbi.nlm.nih.gov/research/bionlp/Tools/sr4gn/
FuzzySearch	https://github.com/maxbachmann/RapidFuzz
**Pre-trained language model**	
arboEL	https://github.com/dhdhagar/arboEL
GenBioEl	https://github.com/Yuanhy1997/GenBioEL
BioSyn	https://github.com/dmis-lab/BioSyn

Table 4. Overview of the biomedical entity linking systems benchmarked on BELB.

In Table 4 we report all systems (and the link to their original implementation) taken into consideration in our benchmarking.

F.1. Rule-based entity-specific systems

	Entity type(s)	Recognition	Linking
GNormPlus	Gene	CRF	Inference Network + TF-IDF + Rules
TaggerOne	Disease, Cell line	semi-Markov model	TF-IDF
BC7T2W	Chemical	-	String matching + Embeddings
SR4GN	Species	String matching	String matching + Rules
tmVar	Variant	String matching	String matching + Rules
SciSpacy	UMLS	-	TF-IDF
FuzzySearch	-	-	Levenshtein Distance

Table 5. Overview of the rule-based entity-specific baselines benchmarked on BELB.

In Table 5 we provide an overview of the rule-based entity-specific models. Implementation details for each model are reported below.

GNormPlus uses a CRF Model to perform entity recognition. The normalization component is a statistical inference network based on TF-IDF frequencies. The system comes with two pre-trained models, namely: "GNR.Model", which was trained on the train and development split (as defined by BELB) of `GNormPlus` and "GNR.GNormPlusCorpus_NLMGeneTrain.Model", which was trained on the whole `GNormPlus` corpus and the train and development split (as defined by BELB) of the `NLM-Gene` corpus. We use the first one when evaluating on `GNormPlus` and the second on `NLM-Gene`.

SR4GN (Species Recognition for Gene Recognition) is a rule-based system which, as the name suggests, is mainly a support component for gene normalization. It implements several custom rules to address cases where species information is not explicitly available. We run GNormPlus as well on the species corpora as SR4GN is only available as a GNormPlus component.

tmVar uses pattern matching to both recognize and normalize variants, falling back to dictionary-lookup when the first fails. It is distributed with a pre-trained model and a pre-processed version of dbSNP. Its usage depends on GNormPlus, as it requires normalized gene mentions to perform linking.

TaggerOne is a general purpose joint recognition and linking system based on a semi-Markov model. It provides two models: "model_NCBID.bin" and "model_BC5CDRD.bin" which are trained on the train split of `NCBI Disease` and the disease annotations of

BC5CDR respectively. Wei et al. (2019) reports to use a TaggerOne model trained on BioID. They however do not publicly release the trained model. Our attempt to use TaggerOne training code resulted in a error.
BC7T2W is a hybrid model based on dictionary-lookup and BioBERT embeddings (Lee et al., 2020). By default the systems includes in the target KB all mentions in NLM-Chem with their gold labels. We modify the "settings.yaml" file to disable this option.
SciSpacy comes with a joint entity recognition and linking pipeline. However, it is possible to run only the linking component using the abbreviation detection module separately (we use the one integrated in the "en_core_sci_sm" model[4]) and feeding the result to linking system ("scispacy_linker").
FuzzySearch This approach assigns a score in $[0, 1]$ to pairs of strings. The score is defined as $\frac{D}{|s_i|+|s_j|}$, where D is the edit distance between strings s_i and s_j and $|s_i|$ is the number of characters in s_i. We use the implementation provided by https://github.com/maxbachmann/RapidFuzz. Given a test mention we compute the similarity score with all synonyms in a KB and select the one with the highest score.

F.2. Pre-trained language models

We convert all corpora and KBs in BELB in the format required by each model and re-train them with the default hyper-parameters reported by the original implementations. All models are trained and evaluated using two Nvidia Tesla A100.
BioSyn We use the default hyper-parameters provided by the authors. Unlike the original approach, we exclusively train on the train split of corpora. The backbone pre-trained language model is BioBERT.
GenBioEl is initialized with BART ("large") weights (Lewis et al., 2020). It uses different values for learning rate and warm-up steps for NCBI Disease and BC5CDR. We cannot perform a full hyper-parameter search for each corpus, and therefore select the values that work best for both corpora, i.e. a learning reate of $1e-5$ and 500 warm-up steps.
arboEL is initialized with BioBERT. The inference procedure for arboEL is parametrized by the number of k nearest neighbor used to construct the graph (determining which pairs of nodes are connected). The implementation provided by the authors runs the inference trying different $k \in \{0, 1, 2, 4, 8\}$. For fair comparison with other models, we do not perform any hyperparameter optimization and hence report the score for $k = 0$.

G. Additional results

	RBES			BioSyn			GenBioEL			arboEL		
	P	R	F1	P	R	F1	P	R	F1	P	R	F1
**Disease**												
NCBI Disease	0.79	0.76	0.77	0.68	0.64	0.64	0.57	0.57	0.57	0.69	0.66	0.66
BC5CDR (D)	0.86	0.85	0.84	0.77	0.74	0.74	0.68	0.70	0.68	0.80	0.80	0.79
**Chemical**												
BC5CDR (C)	0.89	0.80	0.82	0.78	0.71	0.73	0.66	0.67	0.66	0.85	0.84	0.84
NLM-Chem	0.66	0.58	0.59	0.54	0.48	0.49	0.43	0.44	0.42	0.62	0.58	0.58
**Cell line**												
BioID	0.77	0.73	0.74	0.77	0.74	0.75	0.75	0.75	0.75	0.83	0.82	0.82
**Species**												
Linnaeus	0.93	0.94	0.94	0.76	0.75	0.75	0.36	0.35	0.35	0.58	0.53	0.53
S800	0.86	0.84	0.85	0.76	0.72	0.73	0.66	0.66	0.66	0.86	0.85	0.85
**Gene**												
GNormPlus	0.75	0.73	0.73	OOM	OOM	OOM	0.19	0.20	0.19	0.14	0.14	0.14
NLM-Gene	0.62	0.62	0.61	OOM	OOM	OOM	0.10	0.10	0.09	0.10	0.10	0.10
**Variant**												
SNP	0.93	0.92	0.93	-	-	-	-	-	-	-	-	-
Osiris v1.2	0.84	0.83	0.83	-	-	-	-	-	-	-	-	-
tmVar v3	0.89	0.85	0.86	-	-	-	-	-	-	-	-	-
**UMLS**												
MedMentions	0.42	0.39	0.39	OOM	OOM	OOM	0.46	0.46	0.45	0.43	0.43	0.41

Table 6. Macro-average precision (P), recall (R) and F1 score (F1) of all models on the filtered (Section 2.3.2) BELB test sets. OOM: out-of-memory (>200GB)

[4] https://s3-us-west-2.amazonaws.com/ai2-s2-scispacy/releases/v0.5.1/en_core_sci_sm-0.5.1.tar.gz

	RBES	BioSyn	GenBioEl	arboEL
**Disease**	0.94	0.84	0.89	0.87
NCBI Disease	0.94	0.82	0.87	0.86
BC5CDR (D)	0.94	0.87	0.92	0.88
**Chemical**	0.72	0.72	0.81	0.77
BC5CDR (C)	0.82	0.85	0.95	0.88
NLM-Chem	0.67	0.67	0.75	0.72
**Cell line**				
BioID	0.80	0.81	0.95	0.95
**Species**	0.97	0.91	0.85	0.76
Linnaeus	0.99	0.93	0.81	0.74
S800	0.93	0.88	0.93	0.79
**Gene**	0.81	-	0.05	0.30
GNormPlus	0.86	OOM	0.06	0.36
NLM-Gene	0.75	OOM	0.03	0.23
**Variant**	0.90			
SNP	0.94	-	-	-
Osiris v1.2	0.91	-	-	-
tmVar v3	0.88	-	-	-
**UMLS**				
MedMentions	0.58	OOM	0.41	0.69

Table 7. Performance of all models on the filtered (Section 2.3.2) BELB test set with strict evaluation. All scores are micro-average mention-level recall@1. OOM: out-of-memory (>200GB)

	RBES	BioSyn	GenBioEL	arboEL
**Disease**		0.84	0.88	0.84
NCBI Disease	-	0.81	0.84	0.80
BC5CDR (D)	-	0.85	0.89	0.85
**Chemical**	0.72	0.72	0.81	0.77
BC5CDR (C)	0.82	0.85	0.95	0.87
NLM-Chem	0.67	0.67	0.75	0.72
**Cell line**				
BioID	0.82	0.82	0.96	0.95
**Species**		0.87	0.81	0.76
Linnaeus	-	0.89	0.77	0.75
S800	-	0.83	0.89	0.79
**Gene**		-	0.18	0.28
GNormPlus	-	OOM	0.20	0.35
NLM-Gene	-	OOM	0.10	0.21
**Variant**				
SNP	-	-	-	-
Osiris v1.2	-	-	-	-
tmVar v3	-	-	-	-
**UMLS**				
MedMentions	0.58	OOM	0.57	0.69

Table 8. Performance of all baselines on the full test set of all BELB corpora. All scores are micro-average mention-level recall@1. OOM: out-of-memory (>200GB)

H. Updated corpora

In Table 9 we report the number of changes in entity label in the test split of each BELB corpus. For each gold label associated to an entity mention there can be two types of changes. Either the label has been replaced (Replaced), and in this case it can be updated, or it was removed from the KB (Removed), which makes the label obsolete and the mention not linkable, in which case we exclude the entity mention from the test set.

	Entities		
	Replaced	Removed	Total
`GNormPlus`	-	1	1 (0.03%)
`NLM-Gene`	1	1	2 (0.07%)
`NCBI Disease`	-	11	11 (0.25%)
`BC5CDR (C)`	-	243	243 (4.56%)
`NLM-Chem`	-	36	36 (0.31%)
`Linnaeus`	51	-	51 (3.57%)
`S800`	2	-	2 (0.26%)
`BioID`	-	-	0 (0.0%)
`SNP`	6	5	11 (2.13%)
`Osiris v1.2`	7	-	7 (2.68%)
`tmVar v3`	5	-	5 (0.49%)
`MedMentions`	-	-	0 (0.0%)

Table 9. Overview of the number of changes in entity labels in the test split of each corpus in BELB.

References

D. Agarwal, R. Angell, N. Monath, and A. McCallum. Entity Linking via Explicit Mention-Mention Coreference Modeling. In *Proceedings of the 2022 Conference of the North American Chapter of the Association for Computational Linguistics: Human Language Technologies*, pages 4644–4658. Association for Computational Linguistics, 2022. doi: 10.18653/v1/2022.naacl-main.343.

R. Angell, N. Monath, S. Mohan, N. Yadav, and A. McCallum. Clustering-based Inference for Biomedical Entity Linking. In *Proceedings of the 2021 Conference of the North American Chapter of the Association for Computational Linguistics: Human Language Technologies*, page 2598–2608. Association for Computational Linguistics, 2021. doi: 10.18653/v1/2021.naacl-main.205.

C. Arighi, L. Hirschman, T. Lemberger, S. Bayer, R. Liechti, D. Comeau, and C. Wu. Bio-ID track overview. In *BioCreative VI Challenge Evaluation Workshop*, volume 482, page 376, 2017.

A. Bairoch. The Cellosaurus, a Cell-Line Knowledge Resource. *Journal of Biomolecular Techniques : JBT*, 29:25–38, 7 2018. doi: 10.7171/jbt.18-2902-002.

G. R. Brown, V. Hem, K. S. Katz, M. Ovetsky, C. Wallin, O. Ermolaeva, I. Tolstoy, T. Tatusova, K. D. Pruitt, D. R. Maglott, and T. D. Murphy. Gene: a gene-centered information resource at NCBI. *Nucleic Acids Research*, 43(D1):D36–D42, 2015. ISSN 0305-1048. doi: 10.1093/nar/gku1055.

L. Chen, G. Varoquaux, and F. M. Suchanek. A Lightweight Neural Model for Biomedical Entity Linking. *Proceedings of the AAAI Conference on Artificial Intelligence*, 35(14):12657–12665, 2021. ISSN 2374-3468. URL https://ojs.aaai.org/index.php/AAAI/article/view/17499.

A. P. Davis, T. C. Wiegers, R. J. Johnson, D. Sciaky, J. Wiegers, and C. J. Mattingly. Comparative Toxicogenomics Database (CTD): update 2023. *Nucleic Acids Research*, 51:D1257–D1262, 1 2023. doi: 10.1093/nar/gkac833.

R. I. Doğan, R. Leaman, and Z. Lu. NCBI disease corpus: A resource for disease name recognition and concept normalization. *Journal of Biomedical Informatics*, 47:1–10, 2014. ISSN 1532-0464. doi: 10.1016/j.jbi.2013.12.006.

L. I. Furlong, H. Dach, M. Hofmann-Apitius, and F. Sanz. OSIRISv1.2: A named entity recognition system for sequence variants of genes in biomedical literature. *BMC Bioinformatics*, 9, 12 2008. doi: 10.1186/1471-2105-9-84.

A. Hamosh, A. F. Scott, J. S. Amberger, C. A. Bocchini, and V. A. McKusick. Online Mendelian Inheritance in Man (OMIM), a knowledgebase of human genes and genetic disorders. *Nucleic acids research*, 33(suppl_1):D514–D517, 2005.

R. Islamaj, C.-H. Wei, D. Cissel, N. Miliaras, O. Printseva, O. Rodionov, K. Sekiya, J. Ward, and Z. Lu. NLM-Gene, a richly annotated gold standard dataset for gene entities that addresses ambiguity and multi-species gene recognition. *Journal of biomedical informatics*, 118:103779, 2021. ISSN 1532-0480. doi: 10.1016/j.jbi.2021.103779.

R. Islamaj, R. Leaman, D. Cissel, C. Coss, J. Denicola, C. Fisher, R. Guzman, P. G. Kochar, N. Miliaras, Z. Punske, K. Sekiya, D. Trinh, D. Whitman, S. Schmidt, and Z. Lu. NLM-Chem-BC7: manually annotated full-text resources for chemical entity annotation and indexing in biomedical articles. *Database*, 2022, 12 2022. doi: 10.1093/database/baac102.

T. Lai, H. Ji, and C. Zhai. BERT might be Overkill: A Tiny but Effective Biomedical Entity Linker based on Residual Convolutional Neural Networks. In *Findings of the Association for Computational Linguistics: EMNLP 2021*, page 1631–1639. Association for Computational Linguistics, 2021.

J. Lee, W. Yoon, S. Kim, D. Kim, S. Kim, C. H. So, and J. Kang. BioBERT: a pre-trained biomedical language representation model for biomedical text mining. *Bioinformatics*, 36(4):1234–1240, 2020.

M. Lewis, Y. Liu, N. Goyal, M. Ghazvininejad, A. Mohamed, O. Levy, V. Stoyanov, and L. Zettlemoyer. BART: Denoising Sequence-to-Sequence Pre-training for Natural Language Generation, Translation, and Comprehension. *Proceedings of the 58th Annual Meeting of the Association for Computational Linguistics*, 7 2020. doi: 10.18653/v1/2020.acl-main.703.

J. Li, Y. Sun, R. J. Johnson, D. Sciaky, C.-H. Wei, R. Leaman, A. P. Davis, C. J. Mattingly, T. C. Wiegers, and Z. Lu. BioCreative V CDR task corpus: a resource for chemical disease relation extraction. *Database*, 2016(baw068), 2016. ISSN 1758-0463. doi: 10.1093/database/baw068.

C. E. Lipscomb. Medical subject headings (MeSH). *Bulletin of the Medical Library Association*, 88(3):265, 2000.

F. Liu, E. Shareghi, Z. Meng, M. Basaldella, and N. Collier. Self-Alignment Pretraining for Biomedical Entity Representations. In *Proceedings of the 2021 Conference of the North American Chapter of the Association for Computational Linguistics: Human Language Technologies*, page 4228–4238. Association for Computational Linguistics, 2021. doi: 10.18653/v1/2021.naacl-main.334.

G. Martin, N. Goran, and B. Casey M. LINNAEUS: A species name identification system for biomedical literature. *BMC Bioinformatics*, 11, 2010. ISSN 1471-2105. doi: 10.1186/1471-2105-11-85.

S. Mohan and D. Li. MedMentions: A Large Biomedical Corpus Annotated with UMLS Concepts. In *In Proceedings of the 2019 Conference on Automated Knowledge Base Construction (AKBC 2019)*, 2019.

A. A. Morgan, Z. Lu, X. Wang, A. M. Cohen, J. Fluck, P. Ruch, A. Divoli, K. Fundel, R. Leaman, J. Hakenberg, C. Sun, H.-h. Liu, R. Torres, M. Krauthammer, W. W. Lau, H. Liu, C.-N. Hsu, M. Schuemie, K. B. Cohen, and L. Hirschman. Overview of BioCreative II gene normalization. *Genome Biology*, 9:S3, 2008. doi: 10.1186/gb-2008-9-s2-s3.

E. Pafilis, S. P. Frankild, L. Fanini, S. Faulwetter, C. Pavloudi, A. Vasileiadou, C. Arvanitidis, and L. J. Jensen. The SPECIES and ORGANISMS resources for fast and accurate identification of taxonomic names in text. *PLOS ONE*, 8(6):e65390, 2013. ISSN 1932-6203. doi: 10.1371/journal.pone.0065390.

A. S. Schwartz and M. A. Hearst. A simple algorithm for identifying abbreviation definitions in biomedical text. In *Biocomputing 2003*, pages 451–462. World Scientific, 2002.

F. Scott. The NCBI Taxonomy database. *Nucleic Acids Research*, 40:D136–D143, 1 2012. ISSN 0305-1048. doi: 10.1093/nar/gkr1178.

S. Sohn, D. C. Comeau, W. Kim, and W. J. Wilbur. Abbreviation definition identification based on automatic precision estimates. *BMC Bioinformatics*, 9, 12 2008. doi: 10.1186/1471-2105-9-402.

M. Sung, H. Jeon, J. Lee, and J. Kang. Biomedical Entity Representations with Synonym Marginalization . In *Proceedings of the 58th Annual Meeting of the Association for Computational Linguistics*, pages 3641–3650. Association for Computational Linguistics, 2020. doi: 10.18653/v1/2020.acl-main.335.

P. Thomas, T. Rocktäschel, J. Hakenberg, Y. Lichtblau, and U. Leser. SETH detects and normalizes genetic variants in text. *Bioinformatics*, 32(18):2883–2885, 2016. ISSN 1367-4803. doi: 10.1093/bioinformatics/btw234.

P. E. Thomas, R. Klinger, L. I. Furlong, M. Hofmann-Apitius, and C. M. Friedrich. Challenges in the association of human single nucleotide polymorphism mentions with unique database identifiers. *BMC Bioinformatics*, 12, 12 2011. doi: 10.1186/1471-2105-12-s4-s4.

E. Tutubalina, A. Kadurin, and Z. Miftahutdinov. Fair Evaluation in Concept Normalization: a Large-scale Comparative Analysis for BERT-based Models. In *Proceedings of the 28th International Conference on Computational Linguistics*, page 6710–6716. International Committee on Computational Linguistics, 2020. doi: 10.18653/v1/2020.coling-main.588.

M. Varma, L. Orr, S. Wu, M. Leszczynski, X. Ling, and C. Ré. Cross-Domain Data Integration for Named Entity Disambiguation in Biomedical Text. *Findings of the Association for Computational Linguistics: EMNLP 2021*, 11 2021. doi: 10.18653/v1/2021.findings-emnlp.388.

C.-H. Wei, H.-Y. Kao, and Z. Lu. GNormPlus: An integrative approach for tagging genes, gene families, and protein domains. *BioMed Research International*, 2015:e918710, 2015. ISSN 2314-6133. doi: 10.1155/2015/918710.

C.-H. Wei, A. Allot, R. Leaman, and Z. Lu. PubTator central: automated concept annotation for biomedical full text articles. *Nucleic Acids Research*, 47(W1):W587–W593, 2019. ISSN 0305-1048. doi: 10.1093/nar/gkz389.

C.-H. Wei, A. Allot, K. Riehle, A. Milosavljevic, and Z. Lu. tmVar 3.0: an improved variant concept recognition and normalization tool. *Bioinformatics*, 38(18):4449–4451, 2022. ISSN 1367-4803. doi: 10.1093/bioinformatics/btac537.

H. Yuan, Z. Yuan, and S. Yu. Generative Biomedical Entity Linking via Knowledge Base-Guided Pre-training and Synonyms-Aware Fine-tuning. In *Proceedings of the 2022 Conference of the North American Chapter of the Association for Computational Linguistics: Human Language Technologies*. Association for Computational Linguistics, 2022. doi: 10.18653/v1/2022.naacl-main.296.

S. Zhang, H. Cheng, S. Vashishth, C. Wong, J. Xiao, X. Liu, T. Naumann, J. Gao, and H. Poon. Knowledge-Rich Self-Supervision for Biomedical Entity Linking. In *Findings of the Association for Computational Linguistics: EMNLP 2022*, pages 868–880. Association for Computational Linguistics, 2022.
